# Evaluation of a state-wide intervention on salt intake in primary schoolchildren living in Victoria, Australia

**DOI:** 10.1017/S1368980023000332

**Published:** 2023-07

**Authors:** Carley A Grimes, Kristy A Bolton, Kathy Trieu, Jenny Reimers, Sian Armstrong, Bruce Bolam, Kelsey Beckford, Joseph Alvin Santos, Emalie Rosewarne, Elizabeth K Dunford, Stephen Jan, Jacqui Webster, Bruce Neal, Caryl Nowson, Mark Woodward

**Affiliations:** 1 Deakin University, Institute for Physical Activity and Nutrition, School of Exercise and Nutrition Sciences, Geelong, VIC 3216, Australia; 2 The George Institute for Global Health, University of New South Wales, Sydney, NSW, Australia; 3 Victorian Health Promotion Foundation (VicHealth), Melbourne, VIC, Australia; 4 Heart Foundation, Melbourne, VIC, Australia; 5 Department of Health and Human Services, Melbourne, VIC, Australia; 6 Deakin University, School of Exercise and Nutrition Sciences, Geelong, VIC, Australia; 7 Department of Nutrition, The University of North Carolina at Chapel Hill, Chapel Hill, USA; 8 The George Institute for Global Health, School of Public Health, Imperial College London, London, UK

**Keywords:** Salt reduction intervention, Public health, Child, Australia

## Abstract

**Objective:**

In 2015, the Victorian Salt Reduction Partnership launched a 4-year multifaceted salt reduction intervention designed to reduce salt intake by 1 g/d in children and adults living in Victoria, Australia. Child-relevant intervention strategies included a consumer awareness campaign targeting parents and food industry engagement seeking to reduce salt levels in processed foods. This study aimed to assess trends in salt intake, dietary sources of salt and discretionary salt use in primary schoolchildren pre- and post-delivery of the intervention.

**Design:**

Repeated cross-sectional surveys were completed at baseline (2010–2013) and follow-up (2018–2019). Salt intake was measured via 24-h urinary Na excretion, discretionary salt use behaviours by self-report and sources of salt by 24-h dietary recall. Data were analysed with multivariable-adjusted regression models.

**Setting:**

Victoria, Australia.

**Participants:**

Children aged 4–12 years

**Results:**

Complete 24-h urine samples were collected from 666 children at baseline and 161 at follow-up. Mean salt intake remained unchanged from baseline (6·0; se 0·1 g/d) to follow-up (6·1; 0·4 g/d) (*P* = 0·36), and there were no clear differences in the food sources of salt and at both time points approximately 70 % of children exceeded Na intake recommendations. At follow-up, 14 % more parents (*P* = 0·001) reported adding salt during cooking, but child use of table salt and inclusion of a saltshaker on the table remained unchanged.

**Conclusion:**

These findings show no beneficial effect of the Victorian Salt Reduction Partnership intervention on children’s salt intake. More intensive, sustained and coordinated efforts between state and federal stakeholders are required.

Globally salt consumption is too high^(1)^. Whilst the WHO recommends adults consume less than 5 g of salt per d^(2)^, the average intake of Australian adults is 9·6 g/d^(3)^. Australian children also over-consume salt, with an average intake of 5·9 g/d; three-quarters exceed age-specific dietary recommendations^(4)^. Over-consumption of salt (i.e. sodium chloride) leads to higher blood pressure (BP) and consequently CVD^(5)^. Evidence from meta-analyses of randomised controlled trials (RCT) in adults shows that reductions in salt intake lead to reductions in BP, in hypertensive and normotensive individuals^(6,7)^. Meta-analyses of prospective cohort studies have linked higher salt intakes with increased risk of stroke and CVD^(8)^ and increased risk of mortality from stroke and CHD^(6)^.

The detrimental effect of excess salt intake on BP and cardiovascular health begins early in life. Among children, meta-analyses of observational studies show that higher salt intake is associated with higher BP and meta-analyses of RCT show reductions in salt intake lower BP^(9)^. This early-life influence of salt intake on BP is important, because BP tracks over the life course and higher BP levels during childhood predict hypertension^(10)^, as well as subclinical damage to the vascular system, in later life^(11)^. Further concerns of excess salt during childhood relate to taste preferences. Primary school-aged children are known to prefer foods containing higher levels of salt^(12)^, and early-life exposure to salty foods is related to the development of salty taste preference^(13)^. For these reasons, it is crucial that interventions to reduce salt intake target children^(14)^.

To tackle high salt consumption and reduce premature mortality from non-communicable diseases, in 2013 WHO member states (including Australia) voluntarily agreed to reduce mean population salt intake by 30 % by 2025^(15)^. Despite this commitment, there has been no coordinated national salt reduction strategy in Australia^(16)^. As a result, in 2014, a state-level partnership (The Victorian Salt Reduction Partnership), was launched in the Australian state of Victoria to reduce mean population salt intake of Victorian adults and children by 1 g/d by 2020^(17)^. The partnership, led by the Victorian Health Promotion Foundation (VicHealth) and comprised of peak public health organisations, developed a 4-year (2015–2019) (extended to 2020) action plan for population salt reduction.

The multifaceted intervention included four interventions: raising consumer awareness; generating public debate; engaging food industry and advocacy and policy strengthening^(17–19)^. Intervention strategies were designed to impact population salt intake, including children and adults, and were informed by previous state- and community-level salt reduction interventions^(20)^. Recognising the role parents play in creating the home food environment and shaping children’s food choices^(21)^, household food providers with children were the primary target audience of the developed public awareness campaign. This campaign aimed to raise awareness about reducing salt intake for better health across the lifespan and the hidden sources of salt in everyday packaged foods and promote changes in behaviour to reduce families’ salt intake. This was combined with food reformulation strategies that sought to lower the amount of salt added to processed foods.

The primary objective of this study was to assess trends in salt intake of primary school-aged children, as assessed by 24-h urinary Na excretion, pre- and post-implementation of a state-wide salt reduction intervention. Secondary objectives were to assess trends in reported discretionary salt use behaviours (table, cooking and salt shaker placed on the table) and the sources of salt in the diet. This study sits within a comprehensive evaluation of the Victorian Salt Reduction Partnership strategy^(18,19,22–24)^.

## Methods

### Study design and recruitment of participants

The study design was repeated cross-sectional surveys. Baseline data come from a previously collected survey conducted in primary school-aged children in 2010–2013^(4,25)^. The post-intervention follow-up survey was conducted from 2018 to 2019. Ethical approval was obtained from the Deakin University Ethics Committee (ID numbers: EC62-2009 and HEAG-H 01_2018), the Victorian Department of Education and Early Childhood Development (ID number: 2011_001151), and the Victorian Department of Education and Training (ID number: 2018_003666). Written consent was obtained by the school principal and the primary caregiver; the child provided written assent. Participants were recruited from government and non-government primary schools located throughout Victoria which were identified via online school locators. At baseline, a convenience sample of schools was selected^(4)^. At follow-up, we aimed to recruit 500 children who were comparable to the baseline sample for type of school attended (non-government (32 %) *v*. government (68 %)) and socio-economic disadvantage level of the school, based on school postcode and corresponding Socio-Economic Indexes for Areas (SEIFA) (14 %, 16 % and 70 % of participating schools from the bottom, mid and top SEIFA tertiles) (see online Supplemental Fig. 1). To achieve this, schools with at least ten consenting students at baseline were invited to take part at follow-up (*n* 36 schools). Once exhausted, additional schools were randomly invited and recruitment was targeted to SEIFA tertiles to match the baseline profile of schools.

### Sample size calculation

Four hundred children with a complete 24-h urine collection at baseline and follow-up would provide >90 % power at *α* = 0·05 to detect a ≥ 1 g/d difference in mean salt intake. This assumed a mean salt intake of 6 g/d (sd 2·8 g/d) and an intra-class correlation coefficient of 0·03 for children recruited within schools^(4)^. At follow-up, we aimed to recruit 500 children. This accounted for a 5 % dropout rate and 15 % of samples returned as incomplete, as seen in the baseline survey^(4)^. As we did not reach the desired sample size, a *post hoc* power calculation was completed to help interpret study findings. The *simr* package in R was used to estimate the power of our final model to detect a 1 g/d difference in mean salt intake over time (adjusted for the effects of other factors included in the model, and data clustering within the final sample of 666 at baseline and 161 at follow-up). Based on ten simulations, the derived power was >90 %.

### Salt reduction intervention overview

The intervention was implemented by VicHealth, the Heart Foundation and The George Institute for Global Health, with oversight from the Victorian Salt Reduction Partnership^(18)^. Details can be found elsewhere^(18,19,23,24)^. Key intervention strategies designed to lower salt intake among children included (i) food industry engagement and (ii) a public awareness campaign that targeted parents who were household food providers with children aged 0–12 years. The Heart Foundation met with food manufacturers and retailers and developed tools (e.g. ‘Reformulation Readiness Guide; a best practice guide to salt reduction for Australian manufacturers’) and resources (e.g. case studies documenting reformulation) to support the food industry to reduce salt in packaged foods^(18)^. The public awareness campaign was delivered during 2016–2019 and included ‘Don’t Trust Your Taste Buds’ (June–July 2016) and ‘Unpack the Salt’ (August 2017 to April 2019^(26)^. Key messages of the campaign related to high salt consumption within the Victorian population, including: children and families; the link between excess salt and adverse health outcomes across the lifespan; and the hidden sources of salt in packaged foods. Dissemination methods included a campaign website and digital advertisements (e.g. online banner adverts and social media (Facebook, Twitter and Instagram)). Resources designed to help families lower their salt intake (e.g. low salt recipes; salt swaps guide; tips for reading food labels, herb and spice cooking guide; and blogs written by a dietitian) were available via the campaign website and promoted via digital advertising. Campaign resources promoted a greater consumption of fresh, unprocessed foods that contain no added salt (e.g. fruits and vegetables). Publicity among the target group was also gained via media releases covering topical back to school stories.

### Primary and secondary outcomes

The primary outcome was difference in salt intake assessed by 24-h urinary Na excretion pre- and post-implementation of the Victorian Salt Reduction Partnership intervention. Secondary outcomes included differences in reported discretionary salt use behaviours and dietary sources of salt.

### Demographic characteristics and anthropometry

The primary caregiver completed a survey on demographics including child’s date of birth and sex; and parental educational attainment which was grouped as either low: includes those with some or no level of high school education, mid: includes those with a technical/trade certificate or high: includes those with a university/tertiary qualification. A secondary marker of SES was derived from the child’s school postcode and corresponding SEIFA index. Based on this, children were grouped by tertiles of socio-economic disadvantage. The child’s weight and height were measured using standard protocols^(4)^. Age- and sex-adjusted BMI *Z*-scores were calculated using the 2000 US Centers for Disease Control and Prevention growth charts^(27)^. Children were grouped into weight categories using the International Obesity Task force BMI cut-offs^(28)^.

### 24-h urine collection

The 24-h urine collection is described elsewhere^(4)^. Briefly, children could collect urine on a convenient day (e.g. school day or non-school day defined as weekend, public holiday or school holidays). Urine samples were analysed at a commercial pathology laboratory (Dorevitch Pathology, Melbourne, Australia) for Na, K, creatinine and total volume. All measures were standardised to a 24-h duration collection, that is, (24 h/reported urine duration (hours) x urinary measure. Previously published criteria were used to determine if urine samples were deemed incomplete^(4)^.

### 24-h dietary recall

One 24-h dietary recall was completed in those aged ≥8 years^(4)^. Research staff administering recalls were trained in data collection methods by an Accredited Practising Dietitian. At baseline, a 3-pass 24-h dietary recall was conducted face to face with children^(4)^. At follow-up, data collection was streamlined by utilising a newly developed web-based 5-pass 24-h dietary assessment tool, i.e. the ASA-24-Australia-2016^(29)^. The recall was researcher administered with the child to be consistent with baseline data and recommendations from the developer for this age group. Reported food and beverage intake was converted to nutrient intakes using food composition database AUSNUT 2011–2013. Implausible intakes of energy were assessed by comparing each child’s ratio of reported energy intake to estimated BMR (EI:estBMR) to the paediatric-adjusted Goldberg cut-off value^(4,30)^, for example, EI:estBMR ratio <0·87 for boys aged 8–12 years and <0·84 for girls aged 8–12 years.

### Discretionary salt behaviours

Questions for the primary carer were ‘Do you add salt during cooking?’, ‘Do you place a salt shaker on your table at meal times?’ and ‘Does your child add salt to their meal at the table or during sandwich preparation?’ Children were asked ‘Do you add salt to your meal at the table?’ For analysis, responses were dichotomised as either ‘yes, usually and yes, sometimes’ *v*. ‘no’, ‘don’t know’ responses were excluded.

### Data analysis

Analyses were obtained using STATA SE (version 15, StataCorp., LP). Data are reported using descriptive statistics ((mean, se or 95 % CI) or (number, percentage (%), 95 % CI). Conversions from Na to salt were based on the molecular weights of Na (23 g/mol) and sodium chloride (58·5 g/mol). The variation in 24-h urinary Na excretion at the school level was 2 % (intra-class correlation coefficient = 0·02) at baseline and 6 % (intra-class correlation coefficient = 0·06) at follow-up. Differences in demographic characteristics between time points were assessed using independent *t* tests or Pearson’s *χ*^2^ tests. To account for clustering of students within schools, all regression models used robust standard errors. Differences in salt intake across time points were assessed with linear regression and adjusted for covariates previously associated with salt intake, for example, age^(25)^, sex^(25)^, SES^(31)^, BMI *Z*-score^(32)^ and day of urine collection (school day *v*. non-school day)^(25)^. The child’s school postcode and corresponding SEIFA index was used as the maker of socio-economic disadvantage in adjusted models. To determine if the effect of the intervention on salt intake differed by sex, age group (4–8 years; 9–13 years) or SES (parental educational attainment or socio-economic disadvantage defined by school postcode), we examined interaction terms between each of these variables and survey time points and present stratified findings. Logistic regression was used to examine differences, from baseline to follow-up, in: (i) the percentage of children exceeding the age-specific upper level for Na intake (i.e. 2000 mg/d for 4–8 years and 2300 mg/d for 9–13 years)^(33)^, with adjustment for covariates as above; and (ii) discretionary salt use behaviours with adjustment for age, sex and socio-economic disadvantage. For all models, post-estimation was used to derive adjusted proportions. Differences in energy intake (kJ/d), Na intake (mg/d) and salt equivalent (g/d), obtained from 24-h dietary recall were assessed with linear regression models adjusted for age, sex, socio-economic disadvantage, BMI *Z*-score and day of diet recall (school day *v*. non-school day). The population proportion method^(34)^ was used to calculate the contribution of Na from food groups, and those that contributed to ≥1·5 % of Na intake are reported. The contribution of Na from ‘core’ and ‘discretionary’ foods as defined by the Australian Guide to Health Eating^(35)^ is reported. Understanding shifts in Na from these types of foods can inform future strategies required to lower Na intake. Independent *t* tests were used to assess changes in the contribution of Na consumed from different food groups. A *P*-value of <0·05 was considered significant.

## Results

A participation recruitment flow chart is shown in Supplemental Fig. 1. At baseline, of the 509 schools invited, forty-nine agreed to participate (school response rate 10 %). Within participating schools, 14 509 children were invited to participate, of which 852 agreed (child response rate 5 %). Following the exclusion of thirty-one children and forty-one dropouts, 780 children participated. At follow-up, of the fifty-one schools invited to participate, twelve agreed (school response rate 24 %). Within these schools, of the 3030 children invited to participate, 294 agreed (child response rate 10 %). Forty four of these children, recruited across three schools, were excluded as data collection ceased due to the commencement of COVID-19 lockdowns in Victoria in 2020. A further twenty-one children dropped out of the study, leaving 229 children. All follow-up surveys were collected in 2018–2019, prior to the COVID-19 pandemic. The final number of children with a complete 24-h urine collection was 666 at baseline and 161 at follow-up. In brief, *n* 90 children at baseline (12 % of returned urine samples) and *n* 48 children (23 % of returned urine samples) at follow-up provided an incomplete 24-h urine sample and were excluded from analysis. A further three children (*n* 1 at baseline; *n* 2 at follow-up) identified as outliers for 24-h urinary Na excretion were excluded (see online Supplemental Fig. 2).

### Demographic characteristics of participants with a complete 24-h urine sample

Across time points, children were similar with regard to the distribution of sex (just over half were boys) and the type of school attended (about two-thirds attended a government school); however, there were differences with regard to age, socio-economic measures, weight category and whether children completed the 24-h urine sample on a school or non-school day. Specifically, compared with baseline, children at follow-up were older, more were of a healthy weight, more completed the urine collection on a non-school day, more attended a school in an area of lower socio-economic disadvantage and fewer had a parent with a lower level of education (Table 1).

**Table 1:** Demographic characteristics of participants aged 4–12 years with a complete 24-h urine collection at baseline and follow-up

Characteristic	Baseline (*n* 666)		Follow-up (*n* 161)		*P*-value*
	*n* or mean	% or se	*n* or mean	% or se	
Age (years)	9·3	0·1	9·9	0·4	<0·001
Age group					
4–8 years	283	43	48	30	0·003
9–12 years	383	57	113	70	
Sex					
Boy	365	55	84	52	0·55
Girl	301	45	77	48	
Parental educational attainment†					
Low	137	24	18	13	0·01
Mid	85	15	26	18	
High	348	61	99	69	
Socio-economic disadvantage‡					
Bottom tertile (most disadvantaged)	108	16	51	32	<0·001
Mid tertile	148	22	62	39	
Top tertile (least disadvantaged)	410	62	48	30	
School type					
Non-government	227	34	51	32	0·56
Government	439	66	110	68	
Weight category§					
Underweight	67	10	8	5	0·09
Healthy weight	487	73	132	82	
Overweight	92	14	16	10	
Obese	20	3	5	3	
BMI *Z*-score‖	0·10	1·05	0·17	0·91	0·43
Day of urine collection					
School day	317	48	62	39	0·04
Non-school day	349	52	99	62	

* *P*-value determined via Pearson’s *χ*^2^ test or independent *t* test.

† Missing data at baseline *n* 96, follow-up *n* 19.

‡ Based on school postcode and corresponding Socio-Economic Indexes for Areas, Index of Relative Socioeconomic Disadvantage.

§ Weight classification based on the International Obesity Task Force BMI reference cut-offs^(28)^.

‖ BMI *Z*-scores calculated using the 2000 US Centres for Disease Control and Prevention (CDC) Growth Charts as the reference population^(27)^.

### Difference in salt intake assessed by 24-h urinary sodium excretion

There was no difference in mean salt intake assessed by 24-h urinary Na excretion from baseline (6·0; SE 0·1 g/d) to follow-up (6·1; 0·4 g/d) (*P* = 0·81). This finding remained after adjustments (mean difference −0·3 (95 % CI (−0·9, 0·3)) g/d, *P* = 0·36). Similarly, there was no difference in the percentage of children that exceeded the age-specific daily upper level for Na intake (baseline 72 % (95 % CI (69, 75)) *v*. follow-up 70 % (95 % CI (63, 77)); *P* = 0·63; adjusted for covariates). In adjusted analysis, there was evidence to indicate that the effect of the intervention on salt intake differed by sex (*P* = 0·04) and parental level of education (*P* = 0·02) (Table 2). Among boys, there was no difference in salt intake from baseline to follow-up; however, there was a significant −0·6 g/d difference among girls. Among children with a parent of low education level, there was a significant -1·4 g/d difference in salt intake. In contrast, the intervention had no differential effect on salt intake according to age group (*P* = 0·64) or relative socio-economic disadvantage of their school (*P* = 0·57).

**Table 2:** Salt intake (g/d) as assessed by 24-h urinary sodium excretion among participants aged 4–12 years at baseline (*n* 666) and follow-up (*n* 161), stratified by sex, age group, parental educational attainment and socio-economic disadvantage of school

Demographic group	Baseline			Follow-up			Unadjusted			Adjusted*			*P*-value for interaction between demographic characteristic and time point
	*n*	Mean	95 % CI	*n*	Mean	95 % CI	Mean difference	T2, T1	*P*-value	Mean difference	T2, T1	*P*-value	
Sex													
Boys	365	6·4	6·0, 6·7	84	6·8	5·8, 7·7	0·4	−0·5, 1·3	0·39	0·1	−0·7, 0·9	0·79†	**0·04**
Girls	301	5·7	5·4, 6·0	77	5·5	4·6, 6·3	−0·2	−0·9, 0·6	0·65	−0·6	−1·1, -0·2	**0·004** †	
Age group													
4–8 years	283	5·3	5·0, 5·5	48	5·1	4·0, 6·3	−0·2	−1·2, 0·9	0·80	−0·1	−0·8, 0·4	0·55‡	0·64
9–12 years	383	6·6	6·3, 6·9	113	6·6	5·7, 7·4	−0·0	−0·9, 0·8	0·91	−0·3	−1·1, 0·4	0·41†	
Parental educational attainment													
Low	137	6·4	6·0, 6·9	18	5·4	4·2, 6·6	−1·0	−2·2, 0·2	0·09	−1·4	−2·5, -0·3	**0·02** §	**0·02**
Mid	85	5·8	5·4, 6·3	26	6·1	4·6, 7·5	0·2	−1·2, 1·7	0·75	0·5	−0·7, 1·7	0·38§	
High	348	5·8	5·5, 6·1	99	6·5	5·5, 7·3	0·6	−0·4, 1·5	0·22	0·1	−0·6, 0·8	0·73§	
Socio-economic disadvantage of school													
Low (most disadvantaged)	108	6·3	5·9, 6·7	51	6·5	5·3, 7·7	0·2	−1·3, 1·7	0·76	−0·9	−2·0, 0·2	0·10§	0·57
Mid	148	5·6	5·1, 6·2	62	6·3	4·9, 7·7	0·7	−1·2, 2·6	0·44	0·11	−1·2, 1·4	0·85§	
High (least disadvantaged)	410	6·1	5·8, 6·4	48	5·6	4·7, 6·6	−0·5	−1·4, 0·4	0·26	−0·4	−0·9, 0·1	0·08§	

* All regression models adjust for clustering of participants within schools.

† Linear regression with adjustment for age, BMI *Z*-score, day of urine collection and socio-economic disadvantage of school.

‡ Linear regression with adjustment for sex, BMI *Z*-score, day of urine collection and socio-economic disadvantage of school.

§ Linear regression with adjustment for age, sex, BMI *Z*-score and day of urine collection.

*P*-values <0·05 bolded.

### Discretionary salt use behaviours

**Fig. 1:**
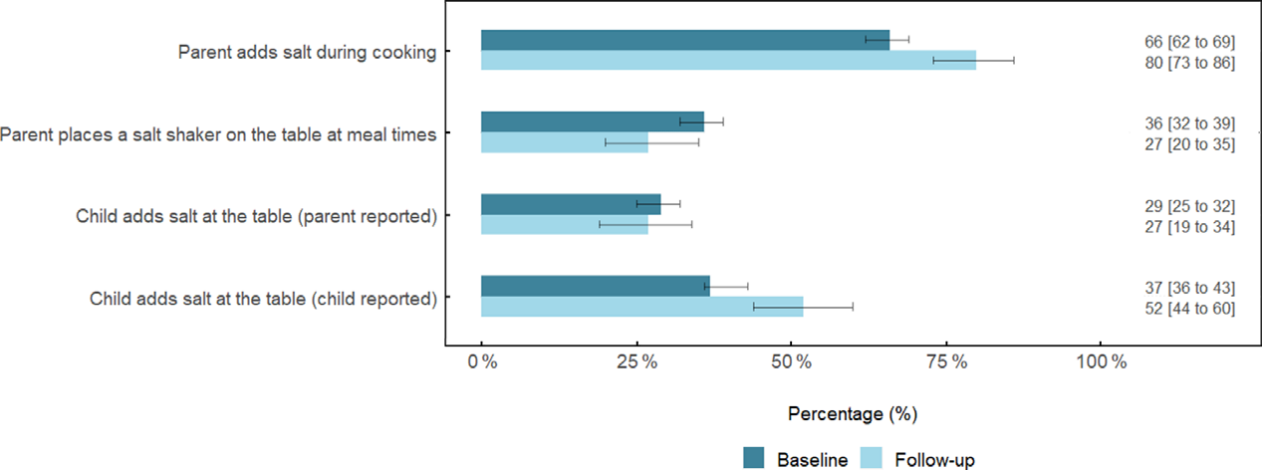
Discretionary salt use behaviours among schoolchildren and their parent at baseline and follow-up^1,2,3^. ^1^Responses represent ‘yes, usually’ and ‘yes, sometimes’. ^2^Values are adjusted for age, sex and school socio-economic disadvantage and clustering of participants within schools. ^3^Data are percentage (%) ± 95 % CI

The percentage of parents who reported adding salt during cooking was 14 % greater (95 % CI (7, 22)) at follow-up compared with baseline (*P* = 0·001) (Fig. 1). There was a non-significant -9 % difference (95 % CI (−17, 0·1)) in the percentage of parents who reported placing a salt shaker on the table at meal times (*P* = 0·06). There was no difference (−2 % (95 % CI (−10, 7)), *P* = 0·69) in the percentage of parents who reported that their child added salt at the table. In contrast, when this behaviour was reported by the child, 14 % (95 % CI (7, 22), *P* = 0·007) more reported adding salt at the table.

### Intake and sources of sodium assessed by 24-h dietary recall

Of the 563 and 181 children who completed a 24-h dietary recall at baseline and follow-up, respectively, 517 (baseline) and 166 (follow-up) were included in the analysis (see online Supplemental Fig. 3). Demographic characteristics of this subset of participants are shown in online Supplemental Table 1. Most dietary recalls captured food intake on a school day; however, this did differ across time points, with more recalls completed on a school day at baseline (79 %) compared with follow-up (62 %) (*P* = 0·001).

**Fig. 2:**
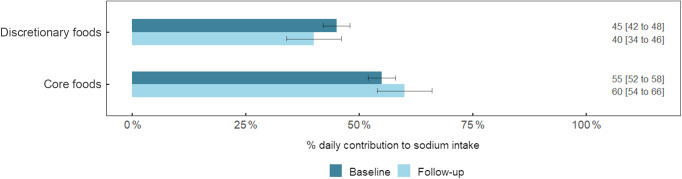
Daily contribution (%) of sodium from ‘core’ and ‘discretionary’ food among participants aged 8–12 years at baseline (*n* 517) and follow-up (*n* 166^)1,2,3,4^. ^1^Core and discretionary foods defined according to the Australian Guide to Healthy Eating^(35,52). 2^The population proportion method was used to calculate contribution of sodium from each food category, for example, (the sum of sodium intake from food category/total sum of sodium from all foods) * 100. ^3^Data are mean ± 95 % CI^. 4^Difference in contribution of sodium from baseline to follow-up for core (*P* = 0·10) and discretionary (*P* = 0·10)

Mean intake of energy (kJ/d), Na (mg/d) and salt equivalent (g/d) at baseline was 8384 (95 % CI (8160, 8608)) kJ/d, 2374 (95 % CI (2293, 2454)) mg/d and 6·0 (95 % CI (5·8, 6·2)) g/d, respectively. Corresponding mean intakes at follow-up were 8552 (95 % CI (7897, 9206)) kJ/d, 2458 (95 % CI (2394, 2702)) mg/d and 6·5 (95 % CI (6·1, 6·9)) g/d. In adjusted models, there were no significant difference between baseline and follow-up intakes of energy (*P* = 0·87) or salt (*P* = 0·05). Overall, there was no shift in the contribution of Na consumed from different food groups across time points (Table 3 and see online Supplemental Fig. 4). The top three sources of Na remained the same, and their contribution to daily intake was regular breads and bread rolls (15 % at baseline and follow-up) mixed dishes where cereal is the major ingredient (10 % at baseline and 15 % at follow-up) and processed meat (8 % at baseline and 7 % at follow-up) (Table 3). The only food groups in which there was a significant difference in their contribution to Na intake across time points were dairy milk (1 % reduction at follow-up; *P* < 0·001), mixed dishes where poultry or feathered game is the major component (4 % increase at follow-up; *P* < 0·001) and potatoes (2 % increase at follow-up; *P* = 0·02) (Table 3). The contribution of Na derived from ‘core’ and ‘discretionary’ foods remained unchanged across time points, core foods contributed to 55–60 % of Na intake and discretionary foods contributed to 40–45 % of Na intake (Fig. 2).

**Table 3:** Daily contribution (%) of sodium from food groups reported at the sub-major food code level among participants aged 8–12 years at baseline (*n* 517) and follow-up (*n* 166)*,†

Food group	Baseline (*n* 517)			Follow-up (*n* 166)			
	Rank	% of daily intake	95 % CI	Rank	% of daily intake	95 % CI	*P*-value‡
Regular breads and bread rolls	1	15	13·2, 16·5	1	15	11·9, 17·5	0·95
Mixed dishes where cereal is the major ingredient§	2	10	8·0, 11·9	2	15	6·7, 22·7	0·19
Processed meat	3	8	6·6, 10·4	3	7	1·4, 11·7	0·03
Cakes, muffins, scones and cake-type desserts	4	5	4·3, 6·0	6	4	1·5, 6·8	0·39
Cheese	5	5	4·0, 5·6	5	4	3·0, 5·9	0·58
Gravies and savoury sauces	6	4	3·4, 5·0	7	4	2·7, 5·5	0·89
English-style muffins, flat breads, and savoury and sweet breads	7	4	2·7, 5·1	8	4	2·1, 5·5	0·91
Pastries	8	4	2·7, 5·0	9	3	1·4, 4·7	0·52
Sausages, frankfurts and saveloys	9	4	2·3, 5·3	11	3	1·6, 4·0	0·30
Breakfast cereals, ready to eat	10	3	2·8, 3·8	13	2	1·5, 3·2	0·05
Dairy milk (cow, sheep and goat)	**11**	**3**	**2·8, 3·5**	**14**	**2**	**1·4, 2·5**	**<0·001**
Savoury biscuits	12	2	1·9, 2·9	10	3	1·5, 4·2	0·46
Soup, home-made from basic ingredients	13	2	1·2, 3·6	17	1	0·2, 2·9	0·29
Mixed dishes where poultry or feathered game is the major component‖	**14**	**2**	**1·6, 2·9**	**4**	**6**	**3·9, 8·0**	**<0·001**
Mixed dishes where beef, sheep, pork or mammalian game is the major component¶	15	2	1·5, 2·6	15	2	0·3, 3·1	0·66
Yeast, and yeast vegetable or meat extracts	16	2	1·4, 2·4	18	1	0·6, 2·4	0·42
Potato snacks	17	2	1·1, 2·4	20	1	0·4, 2·2	0·32
Sweet biscuits	18	2	1·3, 2·0	19	1	0·8, 1·9	0·30
Pasta and pasta products (without sauce)	19	2	1·0, 2·3	21	1	0·4, 1·9	0·24
Potatoes**	**20**	**1**	**0·5, 1·7**	**12**	**3**	**1·3, 3·8**	**0·02**
Poultry and feathered game	21	1	0·1, 0·8	16	2	0·1, 3·0	0·35

* Food groups that provided ≥1·5 % of daily intake of Na at either time point. Food groups ranked in order of greatest contributor to Na intake. Cumulatively all food groups listed contribute to 84 % of Na intake at T1 and T2.

† The population proportion method was used to calculate contribution of Na from each food group, for example, (the sum of Na intake from food group/total sum of Na from all foods) * 100.

‡ *P*-value determined using STATA immediate command for independent *t* tests.

§ Mixed cereal dishes include home-made and commercially available pizza, savoury rice, pasta and noodle dishes, sandwiches and burgers, tacos/tortillas, savoury dumplings and sushi.

‖ Mixed poultry or feathered game dishes includes home-made, frozen and commercially available casseroles, curries, stir-fries, meatballs/rissoles and crumbed/battered products (e.g. chicken nuggets/wings).

¶ Mixed beef, sheep, pork or mammalian game dishes includes home-made, frozen and commercially available casseroles, curries, stir-fries, meatballs/rissoles and crumbed/battered products.

** Potatoes includes home-made and fast-food-style hot potato chips.

*P*-values <0·05 bolded.

## Discussion

This study found no difference in salt intake of primary school-aged children following the implementation of a 4-year multifaceted state-wide intervention to reduce population salt intake. Average salt intake of children remains high at approximately 6 g/d, and three-quarters of children have a salt intake which exceeds the recommended daily upper level^(33)^. During the study period, there was no shift in the food sources of Na within children’s diets and findings related to discretionary salt use were mixed, with some behaviours such as parents use of cooking salt, worsening. In subgroup analysis, we observed a significant reduction in salt intake among girls and children with a parent of lower educational attainment; however, these were in very small sample sizes and need verification in future research. Overall, the findings suggest that the intervention was not effective in shifting children’s salt intake downwards. Some factors that may explain the null finding include the relatively short time frame of the intervention, limited campaign reach, the modest allocated budget (about $750 000 per annum over 5 years, over a population of about 6·6 million within the state of Victoria) and resources, and the reliance on engagement strategies to encourage voluntary food reformulation in combination with education strategies to achieve individual behaviour change.

Independent evaluation on reach of the mass media campaign within a consumer research panel showed that across the duration of the campaign, 27–45 % of the target market (i.e. parents who were primary grocery buyers) recalled at least one element of the salt reduction campaign^(26)^. While this level of campaign reach is comparable to similar Australian government campaigns which have not used television advertising (i.e. 32 % campaign reach), it also indicates that a significant proportion of the target market was not exposed to campaign messages. In the United Kingdom, it took 7 years (2003–2010) of a comprehensive national salt reduction programme to achieve a 1·1 g/d reduction in salt intake among adults (9·5 g/d in 2000/01 to 8·6 g/d in 2011)^(36)^. To date, the programme has not been evaluated in children. This program included (i) a wide-reaching mass media campaign (TV, radio, newspaper and digital advertising), (ii) a reformulation strategy guided by biannually revised voluntary (with threat of legislation) Na content targets set for eighty-five processed food categories, (iii) front-of-pack labelling including salt, and (iv) food procurement policy in specific settings^(36,37)^. The estimated costs of the programme to government over a 10-year period were £92·96 million^(38)^. In comparison, the salt reduction intervention implemented in the state of Victoria was not at the same scale as that conducted in the UK. The success of the UK’s salt reduction programme was predominantly attributable to the reformulation of lower salt foods^(39)^ with a 20–50 % reduction in the salt content of processed foods^(5)^. Changes in consumer behaviour related to choosing lower salt food options were estimated to account for much less of the reduction in salt intake^(39)^.

We found no meaningful changes in the dietary sources of salt following the intervention. Instead, the data indicate that Na intake remains widespread across the food supply, and intake is derived from a wide variety of different food categories. Children obtained significant amounts of dietary Na from both ‘core’ (about 55 %) and ‘discretionary’ (about 45 %) foods. Indicating that to reduce the amount of salt in children’s diets, product reformulation of lower Na ‘core’ foods combined with a suite of strategies designed to shift dietary patterns to align more closely with national dietary guidelines^(40)^ will be required. Progress on reformulation of food products during the intervention was slow^(18)^. The number of food manufacturers that did engage with the partnership on reformulation efforts was relatively small^(18,23)^, and most activities occurred during the second half of the 4-year intervention period, for example, the launch of the ‘Reformulation Readiness Guide – A best practice guide to salt reduction for Australian manufacturers’ occurred in 2019^(18,23)^. A key barrier to achieving progress in this area was the absence of national Na content targets^(18,23)^. It was not until May 2020 that the federal government’s Healthy Food Partnership published the long-awaited national voluntary Na content reduction targets^(41)^. National targets had previously been in place from 2009 to 2013^(42)^; however, due to a change in federal government, these were rescinded. Given this timeline of events, any potential progress made on product reformulation to date from the intervention is unlikely to have been captured during the evaluation period among children, that is, 2018–2019. Furthermore, the relatively modest outcomes achieved with a select number of food manufacturers would be unlikely to significantly drive down children’s salt intake. This is supported by a recent modelling study, which showed that even with complete implementation (100 % compliance) of the updated national voluntary Na reformulation targets on Australian foods, only a very modest reduction in Na (−50 mg/d in Na (0·13 g/d salt); 2·5 % per capita) purchased from packaged foods and beverages would be achieved^(43)^. Some other countries, including South Africa and Argentina, have taken a mandatory approach on Na content targets across a range of processed food items. While this approach has been effective in lowering the Na content of targeted food products, the impact on population Na intake at this point remains unknown^(44)^. The selection of voluntary *v*. mandatory reformulation targets within a Na reduction strategy will be largely dependent on the political setting within the country. In Australia, to achieve success under the current voluntary national programme, there is a need for ongoing strong government leadership, with clear processes and timelines outlined for food industry reporting to ensure accountability to the targets^(44)^. Similar to the voluntary strategy used in the UK, targets should be regularly reviewed and updated to gradually achieve further reductions in Na content, and if compliance is low mandatory legislation should be considered.

The consumer awareness campaign targeted at parents was intended to raise overall awareness about salt in the diet and shift attitudes surrounding the importance of eating less salt. Consequently, this would support changes in behaviour to lower salt in the family diet, such as checking food labels to find lower salt options and not adding salt when preparing foods^(18,19)^. The findings for reported discretionary salt use behaviours were mixed. Two behaviours, parent’s cooking salt and children’s table salt reported by the child, worsened. However, the latter remained unchanged when reported by the parent. The reason for the discrepancy between parent and child reported use of table salt is unclear. There was a trend for a 9 % reduction in parents who reported placing a salt shaker on the table during meal times (*P* = 0·06). This finding is consistent with our previous reports in a larger sample of Victorian parents who were surveyed to more broadly assess changes in salt-related knowledge, attitudes and behaviours (KAB)^(45)^. Specifically, within a previous mid-point evaluation of the intervention, we found that 10 % fewer parents (*P* < 0·001) reported placing a salt shaker on the table during meal times^(45)^, and this change was sustained at the final evaluation follow-up survey (in preparation). However, in contrast to the present study, the mid-point^(45)^ and final evaluation KAB surveys also reported small positive reductions (−9 % and −5 %) in the percentage of parents who reported that their child adds salt at the table and no change in parents’ use of cooking salt. Collectively, findings from these three evaluation studies indicate one message which may have reached parents was the removal of the salt shaker from the table. Other discrepancies in findings between studies may relate to different response rates and methodologies (e.g. recruitment methods) used. Other secular trends external to the intervention may have also contributed to the observed shifts in discretionary salt use behaviours.

There are relatively few studies examining the effectiveness of strategies to reduce salt intake in the paediatric population^(16,46)^. Globally, there is evidence in adults showing the effectiveness of national salt reduction initiatives to reduce population salt intake. The most recent systematic review of these initiatives found that across twenty-five countries, seventeen (70 %) reported a decrease in salt intake^(16)^. However, seven reported no change and one reported an increase. No studies included children, so it is unclear if the same benefits would have reached the paediatric population. It is difficult to tease out the effective components of these multifaceted salt reduction initiatives. To improve understanding in this area, more process evaluations of multifaceted initiatives are required.

Some smaller-scale education-based community-level interventions have been conducted in primary schoolchildren in China, Portugal and Australia. In China, the delivery of a 3·5-month school-based education programme to 5^th^ grade children about the harmful effects of salt and tips to reduce salt intake, with key intervention messages delivered home to parents to influence home food preparation, was shown to be effective in reducing salt intake by 1·9 g/d^(47)^. Similarly in 10–12-year-old children in Portugal, it was shown that a 6-month school-based programme that combined education about the dangers of a high salt diet with a practical school-garden component designed to encourage the use of herbs for salt substitution in home cooking was effective in lowering salt intake by 1·1 g/d^(48)^. On the contrary, in a small study conducted in Victorian primary schoolchildren aged 7–10 years, it was shown that participation in a short 5-week web-based salt reduction behaviour-based education programme, with concurrent participation from parents, was not effective in lowering daily salt intake^(49)^. In summary, while there is some evidence from China and Portugal that school-based education campaigns of reasonable duration (>3 months) that incorporate practical components and a family focus can lower salt intake in children, and both of these countries differ to Australia in that most dietary salt (≈40–60 %) is derived from that which is added at the table and during cooking (i.e. discretionary sources)^(50)^. In Australia, where most salt (about 75 %) consumed comes from processed foods, environmental structural approaches, such as food reformulation and food procurement policies within settings relevant to the paediatric population, such as school canteens, are likely of greater importance. The Victorian Salt Reduction Partnership intended to support and strengthen existing state-based policies related to food procurement in public settings with a focus on embedding salt reduction practices^(19)^. Unfortunately, this outcome was not achieved. Members of the partnership indicated that an absence of relevant stakeholders representing these settings may have impeded any progress in this area^(18,23)^.

The major strength of this study was the use of 24-h urinary Na excretion as an objective marker of dietary salt intake. This method is estimated to capture over 90 % of Na consumed^(51)^. Importantly, this method overcomes limitations associated with alternative dietary recall methods such as misreporting of foods and difficulties in accurately capturing variation in Na content of branded food products. While multiple 24-h urine collections (e.g. three) are required to accurately estimate an individual’s usual Na intake, the use of a single 24-h urine collection is appropriate for estimating average group intake. We did not adjust salt intake for within-person variability; therefore, we likely underestimated the number of children exceeding the upper level for Na intake. A strength of the sampling strategy was the recruitment of children from government and non-government schools located in urban and rural areas within the state of Victoria. At baseline, the final sample was over-representative of children attending schools from a higher socio-economic background, and there were some differences in the demographic profile of children; however, these differences were adjusted for in multivariable analyses. The method used to record reported food intake from interviewer administered 24-h dietary recalls changed from baseline to follow-up, moving from a pen- and paper-based hard copy method at baseline to direct entry into newly available web-based 24-h dietary assessment tool ‘ASA-24’^(29)^. This change helped to streamline data collection and reduce associated study costs. Due to the commencement of COVID-19 lockdowns in Australia, recruitment and data collection ceased earlier than anticipated contributing to the lower number of children with 24-h urine collections and dietary recalls at follow-up. The low response rate at both time points (5–10 %) may introduce non-response bias, and the shortfall in planned numbers at follow-up has reduced the desired precision in results, although the *post hoc* power analysis suggests that the conclusion that a 1 g/d reduction in salt was not achieved is robust. No assessment of parent’s awareness or exposure to the consumer awareness campaign was collected in this study.

In conclusion, this study showed no change in salt intake in this population of primary school-aged children following the implementation of a 4-year state-wide population salt reduction intervention. Acknowledging the low sample size at follow-up, these findings suggest that education strategies targeted at parents combined with relatively limited food industry engagement strategies are inadequate to lower children’s salt intake. More intensive, sustained and coordinated efforts between state and federal stakeholders, including government and food manufacturers/retailers, are required.

## Supplementary material

For supplementary material accompanying this paper visit https://doi.org/10.1017/S1368980023000332. click here to view supplementary material
