# Supplementary material for: Evaluation of a state-wide intervention on salt intake in primary schoolchildren living in Victoria, Australia
Source: Public Health Nutr. 2023 Feb 14;26(7):1456–67. doi: 10.1017/S1368980023000332 (PMC10346046; doi:10.1017/S1368980023000332)
Supplement: Supplementary file 1 [file S1368980023000332sup001.docx]

**Supplementary figure 1.** Recruitment flow chart of participants in the SONIC study at baseline and follow-up^1,2^


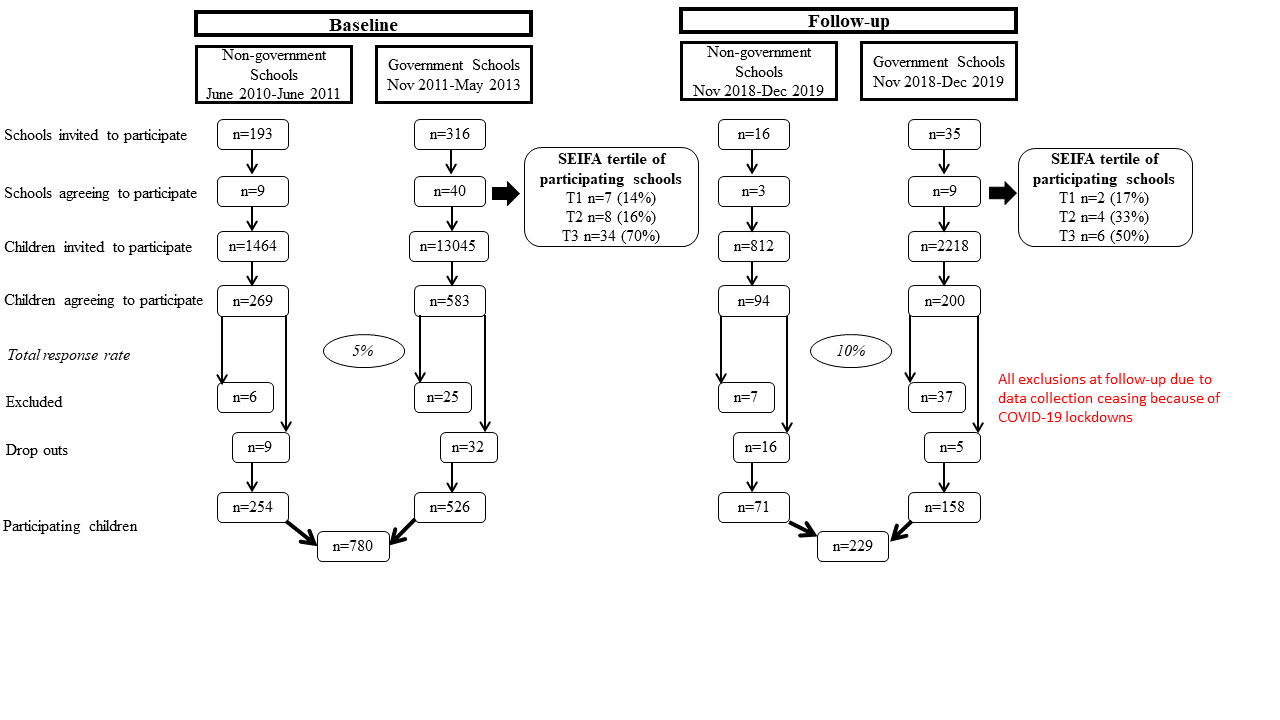


^1^ **Baseline: Non-government schools**

Reasons for exclusion: aged =>13 years (n=6) e.g. in grade 8 of high school

Reasons for attrition: no longer interested in participating (n=3), absent on the day of data collection (n=5), no longer attending the school (n=1)

**Baseline: Government schools**

Reasons for exclusion: n=25 refused to attend data collection day at Deakin University (note: children from participating schools where response rate was low (<10 children) were invited to complete data collection procedures on a scheduled day at Deakin University (e.g. 41 in total invited)

Reasons for attrition: no longer interested in participating (n=7), absent on day of data collection (n=18)

**Follow-up: Non-government schools**

Reasons for exclusion: data collection ceased due to COVID-19 lockdowns (n=7)

Reasons for attrition: no longer interested in participating (n=4), absent on day of data collection (n=9), no longer attending the school (n=3)

**Follow-up: Government schools**

Reasons for exclusion: data collection ceased due to COVID-19 lockdowns (n=37)

Reasons for attrition: absent on day of data collection (n=2), no longer attending the schools (n=3)

^2^ The school’s postcode and corresponding Socio-Economic Indexes for Areas (SEIFA), Index of Relative Socio-Economic Disadvantage was used to group participating schools into tertiles of socioeconomic disadvantage

(T1=bottom tertile, T2=mid tertile, T3=top tertile)

**Supplementary figure 2.** Flow chart for assessment of complete 24-hour urine collections at baseline and follow-up^1^

^
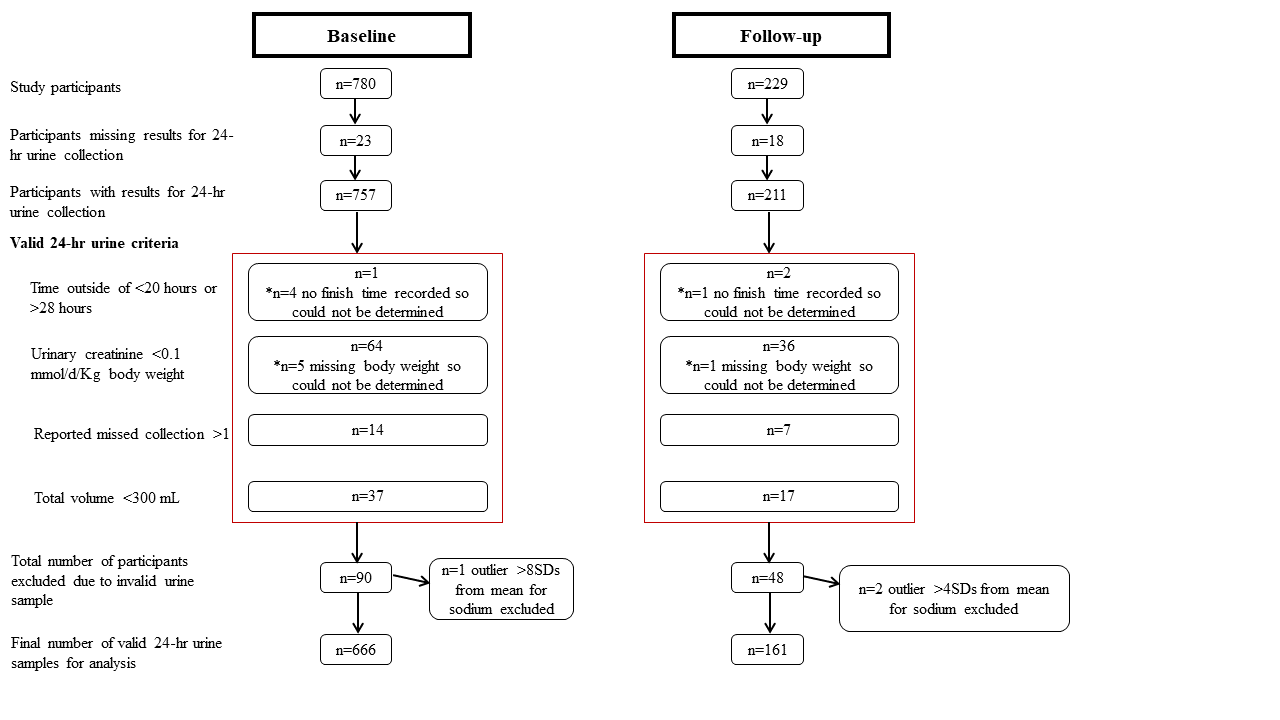
^

^1^Participant’s sample could be excluded on more than one criteria, therefore total number across each of the four criteria exceeds final number of participants excluded

**Supplementary figure 3.** Flow chart for assessment of valid 24-diet recalls at baseline and follow-up


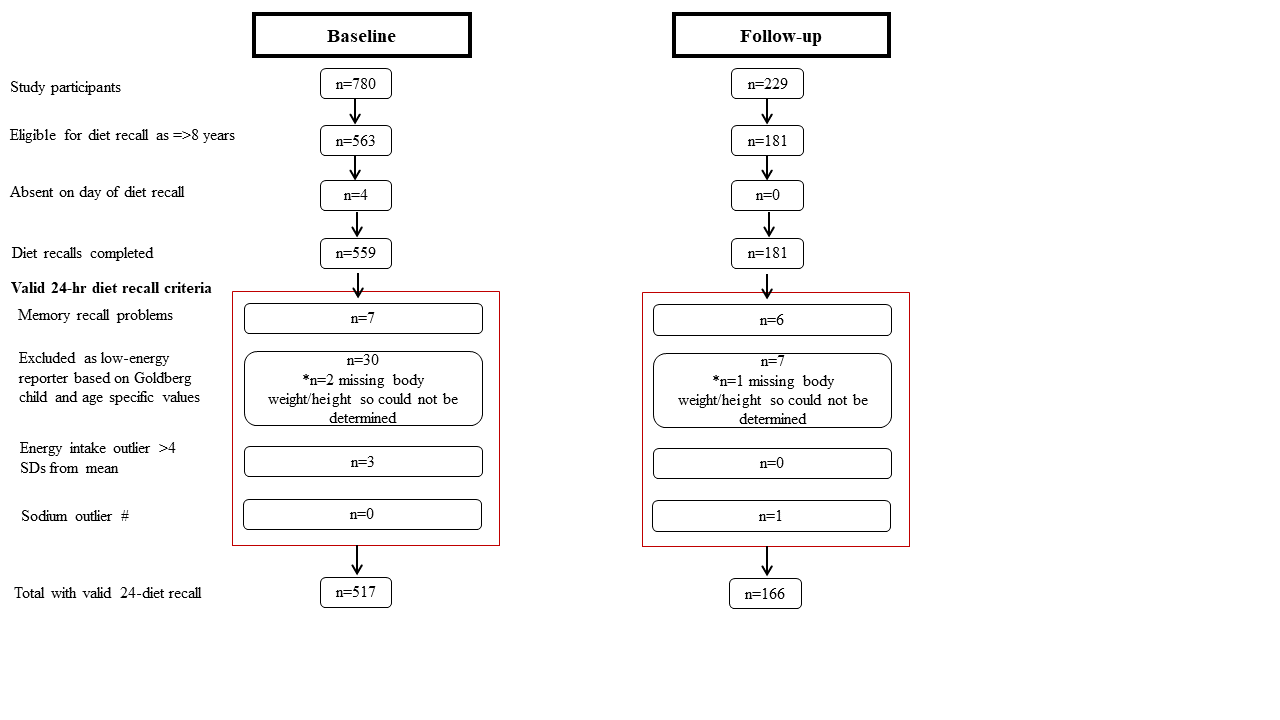


^#^ Follow-up n=1 participant excluded on basis of extreme intake of reported sodium (4 SDs from mean)

**Supplementary figure 4.** Daily contribution (%) of sodium from major food groups among participants aged 8-12 years at T1 (n=517) and T2 (n=166)^1, 2, 3^

^
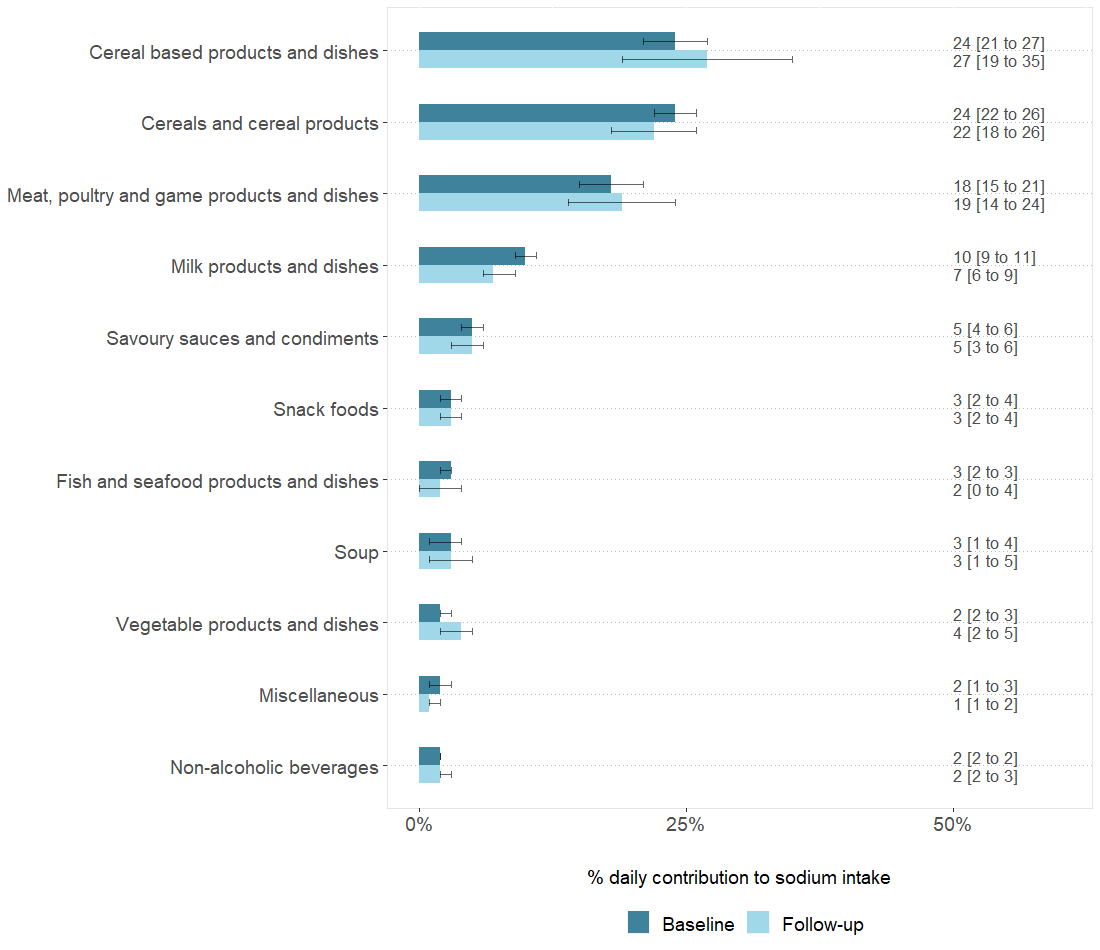
^

^1^ includes major food groups provided ≥1.5% of daily intake of sodium. Food groups ranked in order of greatest contributor to sodium intake.

^2^ The population proportion method was used to calculate contribution of sodium from each food group e.g. (the sum of sodium intake from food group / total sum of sodium from all foods) * 100.

^3^ Data are mean ± 95% confidence interval

**Supplementary table 1.** Demographic characteristics of participants with a valid 24-hour dietary recall

| **Characteristic** | **Baseline (n=517)** | | **Follow-up (n=166)** | | **p-value**^1^ |
| --- | --- | --- | --- | --- | --- |
|  | **n or mean** | **% or SE** | **n or mean** | **% or SE** |  |
| Age (years) | 10.1 | 0.1 | 10.6 | 0.3 | <0.001 |
| Age group |  |  |  |  |  |
| 4-8 years | 120 | 23% | 21 | 13% | 0.003 |
| 9-12 years | 397 | 77% | 145 | 87% |  |
| Gender |  |  |  |  |  |
| Boy | 277 | 54% | 88 | 53% | 0.89 |
| Girl | 240 | 46% | 78 | 47% |  |
| Parental educational attainment^2^ |  |  |  |  |  |
| Low | 106 | 25% | 16 | 11% | 0.002 |
| Mid | 63 | 15% | 23 | 16% |  |
| High | 255 | 60% | 104 | 73% |  |
| Socioeconomic disadvantage of school^3^ |  |  |  |  |  |
| Low | 92 | 18% | 62 | 37% | <0.001 |
| Mid | 102 | 20% | 59 | 36% |  |
| High | 323 | 18% | 45 | 27% |  |
| School type |  |  |  |  |  |
| Non-government | 201 | 39% | 62 | 37% | 0.73 |
| Government | 316 | 61% | 104 | 63% |  |
| Weight category |  |  |  |  |  |
| Underweight | 37 | 7% | 8 | 5% | 0.38 |
| Healthy weight | 373 | 72% | 131 | 79% |  |
| Overweight | 87 | 17% | 22 | 13% |  |
| Obese | 20 | 4% | 5 | 3% |  |
| BMI z-score | 0.22 | 0.05 | 0.20 | 0.09 | 0.87 |
| Day of dietary recall |  |  |  |  |  |
| School day | 409 | 79% | 102 | 62% | <0.001 |
| Non-school day | 108 | 21% | 64 | 39% |  |

^1^ p-value determined via Pearson’s chi-squared test or Independent t-test

^2^ Missing data at baseline n=93, follow-up n=23

^3^ Based on school postcode and corresponding Socio-Economic Indexes for Areas, Index of Relative Socio-Economic Disadvantage^(1)^

^4^ Weight classification based on the International Obesity Task Force BMI reference cut-offs ^(2, 3)^

^5^ d

## References

1. Australian Bureau of Statistics. Socio-Economic Indexes for Areas: ABS; 2013 [Available from: <https://www.abs.gov.au/websitedbs/censushome.nsf/home/seifa>.

2. Cole TJ. Establishing a standard definition for child overweight and obesity worldwide: international survey. BMJ. 2000;320(7244):1-6.

3. Cole TJ, Flegal KM, Nicholls D, et al. Body mass index cut offs to define thinness in children and adolescents: international survey. BMJ. 2007;335(7612):194-7.
